# Evaluation of an intensive workshop on research methods in supportive oncology

**DOI:** 10.1017/S1478951522001432

**Published:** 2024-08

**Authors:** Ashley Leak Bryant, Jessica L. Krok-Schoen, Ewan K. Cobran, Joseph A. Greer, Jennifer S. Temel, William F. Pirl

**Affiliations:** 1 School of Nursing, University of North Carolina at Chapel Hill, Chapel Hill, NC, USA; 2 Cancer Research Training Education Coordination, Lineberger Comprehensive Cancer Center, Chapel Hill, NC, USA; 3 Division of Health Sciences, School of Health and Rehabilitation Sciences, The Ohio State University, Columbus, OH, USA; 4 Division of Epidemiology, Department of Quantitative Health Science, Mayo Clinic College of Medicine and Sciences, Scottsdale, AZ, USA; 5 Department of Psychiatry and Center for Psychiatric Oncology, Massachusetts General Hospital, Harvard Medical School, Boston, MA, USA; 6 Division of Hematology & Oncology, Department of Medicine, Massachusetts General Hospital Cancer Center, Harvard Medical School, Boston, MA, USA; 7 Department of Psychosocial Oncology and Palliative Care, Dana-Farber Cancer Institute, Harvard Medical School, Boston, MA, USA

**Keywords:** Early career, Supportive oncology, Methodology, Outcomes, Workshop, Research

## Abstract

**Objectives.:**

Since 2015, the Harvard Workshop on Research Methods in Supportive Oncology has trained early-career investigators in skills to develop rigorous studies in supportive oncology. This study examines workshop evaluations over time in the context of two factors: longitudinal participant feedback and a switch from in-person to virtual format during the COVID pandemic.

**Methods.:**

We examined post-workshop evaluations for participants who attended the workshop from 2015 to 2021. We qualitatively analyzed evaluation free text responses on ways in which the workshop could be improved and “other comments.” Potential areas of improvement were categorized and frequencies were compiled longitudinally. Differences in participants’ ratings of the workshop and demographics between in-person and virtual formats were investigated with *t*-tests and Chi-square tests, respectively.

**Results.:**

286 participants attended the workshop over 8 years. Participant ratings of the workshop remained consistently high without substantial variation across all years. Three main themes emerged from the “other comments” item: (1) sense of community; (2) passion and empowerment; and (3) value of protected time. Participants appeared to identify fewer areas for improvement over time. There were no significant differences in participant ratings or demographics between the in-person and virtual formats.

**Signifinace of results.:**

While the workshop has experienced changes over time, participant evaluations varied little. The core content and structure might have the greatest influence on participants’ experiences.

## Introduction

Responding to the need to increase the quality of research in supportive oncology and grow its investigator base, we created the Harvard Medical School Workshop on Research Methods in Supportive Oncology (RMSO). This 6-day intensive workshop provides an overview of essential research methods in supportive oncology and mentorship for participants to develop a full study protocol or grant proposal. Since 2015, the workshop has trained almost 300 early-career investigators with funding from the National Cancer Institute. Participants are psychologists, physicians, nurse researchers, doctorally prepared social workers, and other researchers. Details on the development of the workshop and its initial outcomes have been published elsewhere (Pirl et al. 2018).

While the workshop has maintained a core curriculum and format over the years, it has made modifications in response to new developments and priorities in supportive oncology research, feedback from participants and faculty, and environmental challenges (e.g., the COVID pandemic). In this paper, we examine the outcomes of the workshop in the context of changes over the years. First, we examined participant feedback longitudinally for the first 6 years, when the workshop was offered in person. Second, we compared workshop evaluations and characteristics of participants before and after the switch to a virtual format because of the COVID pandemic.

The virtual format prompted modifications that included a streamlining of the curriculum and the creation of virtual community-building techniques. We applied lessons learned from the use of virtual reality in the research to virtual education. Virtual reality is more engaging when it is immersive and involves multiple senses (Gallace et al. 2012; Sanders et al. 2021). We developed a set of interventions and supplies to create an immersive and multisensory communal experience for participants, which include (1) noise-canceling over-the-ear headphones for the Zoom sessions; (2) a scented candle that is lit during all workshop activities to signify a different space; (3) premade meals from the same national service eaten during educational sessions; (4) photos of Boston to display next to the screen or use as virtual backgrounds for social activities during the workshop; and (5) a playlist of inspiring popular music curated by the course faculty.

## Methods

A descriptive, longitudinal design was used to assess the program evaluation across 8 years (2015–2022), 6 when the format of the workshop was in person (2015–2020) and 2 when it was virtual (2021–2022). We compiled information on the participants’ characteristics from their applications and examined the postworkshop evaluation surveys that the participants completed. The workshop is designed for 36 participants. Each year, approximately 38 participants are accepted and a waitlist is created to manage dropouts before the start of the workshop. Participants are psychologists, physicians, nurse researchers, doctorally prepared social workers, and other researchers.

### Workshop applications

To apply for the workshop, participants were required to submit a one-page application, their National Institutes of Health biosketch, and an abstract for a study they would develop into a protocol at the workshop. The one-page application includes demographic information, such as sex, race and ethnicity, and professional discipline.

### Post-workshop evaluation

All participants completed an anonymous evaluation through Survey Monkey at the end of the workshop. The evaluation included items assessing the participants’ satisfaction with the workshop, self-rated research skills, and perceived value of the workshop and its components. The response format was on a scale of 1–4, with 1 being “poor” and 4 being “excellent.” The postworkshop evaluation also asked participants to identify potential areas for improving the workshop through 2 open-ended questions: (1) “What do you think was missing from the workshop?” and (2) “What do you recommend to improve the workshop?” Participants were also asked to write “other comments” they had about their workshop experience. We examined the open-ended responses for potential areas of improvement and “other comments” only during the in-person years of the workshop in order to identify longitudinal patterns.

### Analyses

Descriptive statistics (mean, frequencies, and categories) were compiled using SPSS 27. Significant differences were investigated with *t*-test for continuous variables and Chi-square tests for proportions. Open-ended questions data were analyzed by coinvestigators ALB, JLK-S, and EKC using procedures of thematic analysis (Patton 1990; 2014). This analysis consists of 2 phases. First, open-ended questions data were reviewed for accuracy. Next, an open coding process as well as dominant issues raised by the participants was conducted. The final phase utilized memoing, which used theoretical notes to document key thoughts and assist with further refinement of codes and the development of themes (Strauss and Corbin 1998). Discussions were held among co-investigators to agree upon coding development and final analysis. NVivo 12 (QSR International Inc., Cambridge, MA) was used to facilitate qualitative analysis.

## Results

Between 2015 and 2022, a total of 286 participants attended the annual workshop. The average acceptance rate over the 8 years was 56%. Three years had very late dropouts before the workshop started, which resulted in less than 36 participants. Two years had 37 participants because only one participant dropped out before the workshop. Table 1 shows the demographic characteristics of the workshop participants. The majority of the participants were female (80%) and White (72%). The evaluations of the workshop are summarized in Table 2. Across all 8 years, 286 of the 286 (100%) participants completed writing a full research protocol or proposal during the workshop.

The “other comments” in the post-workshop evaluation further describe the experience of the workshop for participants. Three main themes emerged from the qualitative analysis: (1) sense of community and connection; (2) passion and empowerment; and (3) the value of protected time. The first theme focused on the sense of community and connection experienced by the workshop participants. For example, one participant shared, “Having always felt like a bit of an outsider as an oncologist interested in supportive care, it was great to feel at this workshop like I was surrounded by my people.” Similarly, a participant stated, “The most valuable asset of the workshop is the opportunity for face-to-face time with other researchers in a field where we are otherwise often lonely.” To describe the connections made by attending the workshop, one participant said, “I probably gained the most from the community building,” while another said, “I had time to have many ‘real’ and in-depth interactions with colleagues. I formed several new friendships.”

The second emergent theme was a newly found and/or renewed passion for supportive care as well as a sense of empowerment. One participant felt that the workshop was “a game changer for me!” Another participant stated that the workshop “advanced my commitment to this work,” while another said, “the workshop re-ignited my passion for what I do.” After the completion of the workshop, one participant shared, “I feel empowered and supported going forward with an entirely new skill set and outlook,” and another stated that they “feel much better prepared for grant writing as well as more motivation to continue my research efforts.”

The last emergent theme was the protected time for learning and writing. One participant appreciated “having a week to myself to just work on this grant because I was able to block out other distractions and get work done.” Another participant stated, “I got more done this week than I would have achieved in a month at home.” Similarly, a participant noted, “Without a doubt this was the most useful and productive 5 days as a researcher.” Lastly, there were numerous comments and requests to come back every year. For example, a participant exclaimed, “I am one of the many who want to come back next year!!”

### Feedback from participants over time for in-person format (2015–2020)

**Fig. 1.**
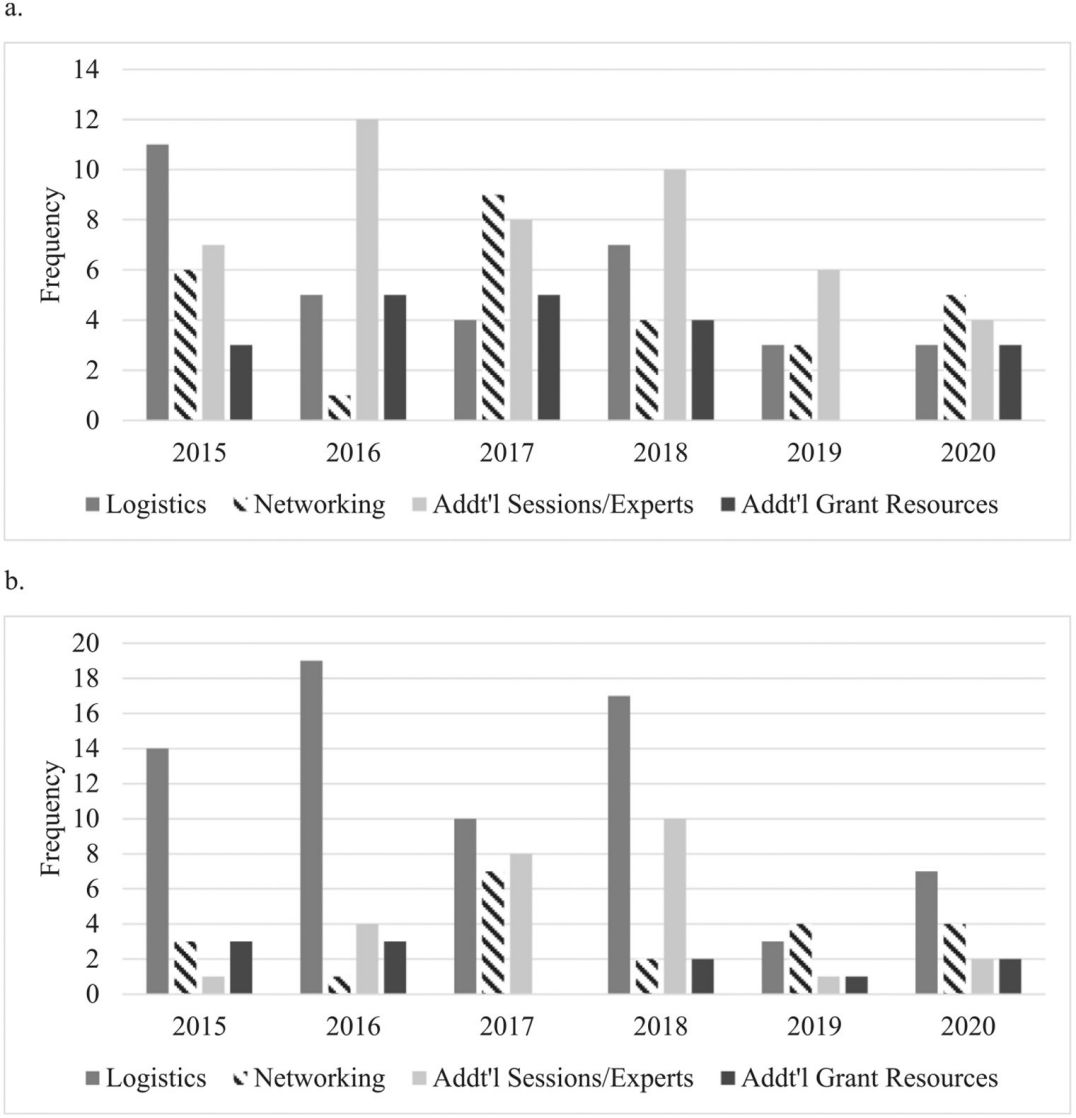
Participant responses regarding missing workshop elements and recommendations by year. (a) Missing workshop elements. (b) Recommendations to improve the workshop.

In the post-workshop surveys, the items identified by participants as missing from the workshop fell into 5 main categories: logistical issues, more networking opportunities, additional sessions or experts, and additional grant resources (Figure 1a). Examples of logistical elements included the need for “bigger rooms” and “written schedules” in addition to an online agenda schedule. Participants suggested additional lectures on a variety of topics such as patient-reported outcomes and greater access to statistical expertise. The number of responses identifying missing elements appeared to decrease over time both overall and within each category.

Figure 1b depicts the frequency and categories of feedback regarding suggested improvements to the RMSO workshop. A number of suggestions were shared by participants to improve the workshop, and these fell into the same categories as for missing elements: logistical issues, more networking opportunities, additional sessions or experts, and additional grant resources. Logistical issues were most frequently cited as ways to improve the works. Similar to the feedback regarding missing workshop elements, the numbers of suggestion for improvement appeared to decrease over time both overall and within each category.

As shown in Table 2, workshop evaluations were consistently positive and similar throughout the first 6 years of the workshop, even though less opportunities for improvement were identified by participants over time.

### Switch to virtual format (2021 and 2022)

There were no significant differences in the demographics of participants who attended the workshop across the years it was in person versus across the years it was virtual. However, even though not statistically significant, less Black/African-American participants attended the virtual workshops compared to the in-person workshops, 14/214 (6.5%) versus 2/72 (2.8%), *X* = 1.54, *p* = 0.23. This is notable as it appears that the number of Black/African-American participants may have been increasing over the years before the switch to virtual.

Participant post-workshop evaluations were consistently positive across all years, regardless of in-person or virtual formats (Table 2). Across the 6 in-person years, the mean rating was 3.92 (SD = 0.32) for satisfaction with the workshop, 3.97 (SD = 0.24) for quality of teaching, 3.89 (SD = 0.35) for value of protocol writing experiences, 3.94 (SD = 0.29) for value of workshop, 3.96 (SD = 0.25) for likelihood of recommending workshop to colleague, and 3.93 (SD = 0.30) for impact on excitement to do supportive oncology research. These did not significantly differ for ratings across the 2 virtual years. For the virtual workshops, the mean rating was 3.96 (SD = 0.21) for satisfaction with the workshop, 4.0 (SD = 0.0) for quality of teaching, 3.91 (SD = 0.29) for value of protocol writing experiences, 3.99 (SD = 0.12) for value of workshop, 3.97 (SD = 0.17) for likelihood of recommending workshop to colleague, and 3.97 (SD = 0.17) for impact on excitement to do supportive oncology research. There were no significant differences.

## Discussion

Intensive research training programs are one way to potentially grow the research workforce and quality of research in supportive oncology. However, to remain successful, training programs must continue to attract a large number of applicants, consistently provide participants with a valuable experience, and adapt to changes in the field to stay relevant over time. We examined evaluation data over 8 years of a 6-day workshop to train earlycareer trainees and faculty on methodological skills in supportive oncology research. Across all years, participants consistently rated the workshop highly in terms of satisfaction, value, quality, and likelihood of recommending it to a colleague.

We observed little variation across years despite changes in the workshop. While suggestions for areas of improvement decreased over time, there did not appear to be a corresponding trend in better evaluations. This might be due to the fact that the evaluations were already quite high and there could be a ceiling effect. Every year, almost all participants rated the evaluation items a 4 on a scale of 1–4, with 4 being “excellent.” The minor variations in items from year to year is the result of the number of participants rating an item as a 3. A scale with greater range might be needed to detect changes over time in response to modifications of the workshop.

The switch from an inperson to a virtual format also did not appear to impact participant evaluations. This is notable given the themes that emerged from the “other comments” during the inperson years of the workshop around community, passion, and protected time. Our virtual community-building techniques may have contributed to still having a sense of community and excitement and the workshop. Virtual participants commented that the workshop felt much different than other virtual programs and that they felt connected to faculty and other participants. While virtual participants were not sequestered in a hotel like the in-person participants, they were still able to complete writing a full research protocol or proposal over the 6 days of the workshop. We had instructed the virtual participants to clear their schedules and responsibilities as much as possible to make it a retreat-like experience, but some still encountered competing demands at home, such as family responsibilities. Previous studies have shown the importance of protected time for clinical researchers to improve their productivity (Barton 2008; Campion et al. 2016; Ryan et al. 2019)and development.

Although there were no significant changes in the demographics of the participants with the switch to the virtual format, we observed lower numbers of Black/African-American participants with the virtual format. When the workshop was in person, the numbers of Black/African-American participants appeared to be increasing over time, with 2019 having 5 Black/African-American participants (including 2 of the co-authors, ALB and EC). Over the 2 years of the virtual format, we have had only 2 Black/African-American participants. This might be related to the disproportionate burden of the COVID pandemic on the Black/African-American community in the US (Vasquez Reyes 2020), which is the reason behind the switch to the virtual format. However, there could be other factors influencing the awareness of the opportunity, its attractiveness, and its feasibility for early-career Black/African-American researchers in the virtual format.

While these data provide some reassurance for converting successful in-person training workshop to virtual formats, we only have short-term data and do not yet have longer-term data on the research success of the participants. It is still unclear whether there may be differences between the in-person and virtual formats for rates of later obtaining research funding or developing productive research collaborations with other participants.

There are also other limitations to this study. The “other comments” section of the workshop evaluation used in the qualitative analyses was not designed specifically to elicit responses on what made the workshop most valuable to participants. These spontaneous comments might not have captured the full range of potential responses. This program also focuses on supportive oncology researchers and their experiences, which might not generalize to other types of researchers doing a similarly structured program.

Other funded programs have been developed and implemented to train and mentor palliative care leaders (Cruz-Oliver et al. 2017; Dahlin et al. 2019; Kamal et al. 2016), oncology clinical investigators(Von Hoff et al. 2021), geriatric nurse leaders and researchers (Bellot et al. 2013; Beverly and Harden 2020; Brody et al. 2016; Bryant et al. 2015; Fagin 2012; Franklin et al. 2011; Harden and Watman 2015), nursing students (Flynn 2015; Gillett et al. 2022) and nurse faculty scholars (Brody et al. 2017; Campbell et al. 2017; Hickey et al. 2014; McBride et al. 2017), but few include an intensive mentored experience with the aim of developing a protocol or grant proposal in a short period of time. The Workshop on RMSO appears to be meeting a need for early-career investigators as evidenced by its demand and its evaluations. While there have been some changes over time, the core elements of the workshop have remained and have consistently led to excellent evaluations and all participants writing a full research protocol or proposal by the end of the workshop. If the workshop is to remain virtual, more attention is required to ensure that it continues to attract early-career investigators from under-represented racial and ethnic groups. We hope that the training from the workshop will continue to produce high-quality interdisciplinary supportive oncology research.

## Figures and Tables

**Table 1.** Participant characteristics (*n* = 286)

Variables	2015 (*n* = 36), *N* (%)	2016 (*n* = 36), *N* (%)	2017 (*n* = 37), *N* (%)	2018 (*n* = 34), *N* (%)	2019 (*n* = 36), *N* (%)	2020 (*n* = 35), *N* (%)	2021 (*n* = 35), *N* (%)	2022 (*n* = 37), *N* (%)
Female sex	26 (72.2)	32 (88.9)	31 (83.8)	26 (76.5)	29 (72.2)	29 (80)	25 (71.4)	31 (83.8)
Race								
White	28 (77.8)	29 (80.6)	26 (70.3)	25 (73.5)	25 (69.4)	20 (55.5)	24 (68.6)	30 (81.1)
Asian	6 (16.7)	3 (8.3)	8 (21.6)	5 (14.7)	6 (16.7)	9 (25)	10 (28.6)	5 (13.5)
African-American/Black	1 (2.8)	2 (5.6)	1 (2.7)	2 (5.9)	5 (13.9)	3 (8.5)	2 (5.6)	0 (0)
Not reported or unknown	2 (5.6)	0 (0)	0 (0)	1 (2.9)	0 (0)	4 (11.4)	0 (0)	2 (5.4)
Ethnicity								
Hispanic or Latino/a	1 (2.8)	2 (5.6)	2 (5.4)	1 (2.9)	4 (11.1)	4 (11.4)	1 (2.9)	4 (10.8)

**Table 2.** Post-workshop participant evaluations

Variables	2015 (*n* = 36), mean (SD)	2016 (*n* = 36), mean (SD)	2017 (*n* = 37), mean (SD)	2018 (*n* = 34), mean (SD)	2019 (*n* = 36), mean (SD)	2020 (*n* = 35), mean (SD)	2021 (*n* = 34), mean (SD)	2022 (*n* = 33), mean (SD)
Satisfaction with workshop	3.97 (0.17)	3.81 (0.40)	3.97 (0.17)	3.94 (0.23)	3.88 (0.54)	3.97 (0.17)	3.94 (0.24)	3.97 (0.17)
Quality of teaching	4.00 (0)	3.97 (0.16)	4.0 (0)	3.97 (0.16)	3.88 (0.54)	4.0 (0.0)	4.0 (0.0)	4.0 (0.0)
Value of protocol writing experience	3.91 (0.29)	3.78 (0.48)	4.0 (0)	3.94 (0.23)	3.88 (0.54)	3.83 (0.38)	3.91 (0.37)	3.91 (0.29)
Value of workshop	3.97 (0.17)	3.83 (0.16)	3.97 (0.17)	3.97 (0.16)	3.91 (0.52)	4.0 (0.0)	3.97 (0.17)	4.0 (0.0)
Likelihood of recommending workshop to a colleague	3.97 (0.17)	3.97 (0.16)	4.0 (0)	3.94 (0.23)	3.90 (0.52)	4.0 (0.0)	3.97 (0.17)	3.97 (0.17)
Impact on excitement to do supportive oncology research	3.94 (0.25)	3.94 (0.23)	3.97 (0.17)	3.92 (0.28)	3.88 (0.54)	3.97 (0.17)	3.97 (0.17)	3.97 (0.17)

Scale: 1 = “poor” to 4 = “excellent.”
